# Feasibility of LifeFul, a relationship and reablement-focused culture change program in residential aged care

**DOI:** 10.1186/s12877-018-0822-3

**Published:** 2018-05-31

**Authors:** Lee-Fay Low, Shruti Venkatesh, Lindy Clemson, Dafna Merom, Anne-Nicole Casey, Henry Brodaty

**Affiliations:** 1 The University of Sydney, Sydney, NSW 2006 Australia; 2 Western Sydney University, Campbelltown, Sydney, NSW 2560 Australia; 3 Centre for Healthy Brain Ageing (CHeBa), the University of New South Wales, Sydney, NSW 2052 Australia

**Keywords:** Nursing home, Residential aged care, Long-term care, Reablement, Restorative care, Relationship-focused care, Person-centred care

## Abstract

**Background:**

The protective, custodial, task-oriented care provided in residential aged care facilitates decreases health and wellbeing of residents. The aim of the study was to conduct a feasibility study of LifeFul – a 12 month reablement program in residential aged care.

**Methods:**

LifeFul was developed based on systematic reviews of reablement and staff behaviour change in residential aged care, and in consultation with aged care providers, consumers and clinicians. LifeFul includes: engaging and supporting facility leaders to facilitate organisational change, procedural changes including dedicated rostering, assigning each resident a ‘focus’ carer and focusing on the psychosocial care of residents part of handovers and staff training. The study was conducted in three Australian residential aged care facilities. A pre-post mixed methods design was used to evaluate recruitment and retention, fidelity and adherence, acceptability, enablers and barriers and suitability of outcome measures for the program.

**Results:**

Eighty of 146 residents agreed to participate at baseline and 69 of these were followed up at 12 months. One hundred and four of 157 staff participated at baseline and 85 of 123 who were still working at the facilities participated at 12 months. Staff perceived the program to be acceptable, barriers included having insufficient time, having insufficient staff, negative attitudes, misunderstanding new procedures, and lack of sufficient leadership support. Quantitative data were promising in regards to residents’ depression symptoms, functioning and social care related quality of life.

**Conclusion:**

It is feasible to deliver and evaluate LifeFul. The program could be improved through increased leadership training and support, and by focusing efforts on residents having a ‘best week’ rather than on completing a document each handover.

**Trial registration:**

Registered prospectively on 22nd January 2016 on ANZCTR369802.

**Electronic supplementary material:**

The online version of this article (10.1186/s12877-018-0822-3) contains supplementary material, which is available to authorized users.

## Background

The protective, custodial and task-oriented model of care provided in residential aged care has been reported to have a deleterious impact on aged care residents, including excessive disability, poor self -care [1, 2], functional decline, decreased physical activity and deconditioning [3]. In contrast, reablement or restorative models of care focuses on the restoration and/or maintenance of function and helps older adults to compensate for impairments with ageing or illness [4]. Restorative care sets each person a specific goal or desired outcome, such as adapting to some functional loss, or regaining confidence and capacity to resume past activities. There is a growing body of evidence that reablement/restorative care practices in residential aged care improve residents’ physical condition and social functioning (e.g. [5–13]).

Preferences of older people support a reablement approach and relationship-focused model of care. Residents have stated that relating to staff is one of the most important aspects of the care they receive [14] and want staff who build relationships with them and their families. Further, residents would like opportunities for rehabilitation, mobility and physical exercise, social interaction and engagement in meaningful leisure activities [15].

Aged care policy has begun to emphasize reablement approaches. Reablement approaches are being trialed in the UK, Netherlands and New Zealand [16]. The Australian Productivity Commission recommended that older Australians receive a flexible range of care and support services that meet their individual needs and that emphasize reablement and rehabilitation [17]. The Commonwealth Home Support Program also has a focus on wellness, reablement and restorative care and seeks to actively promote independence [18].

Successful reablement program components identified in the literature include establishing a new philosophy of care [4, 19], setting individual goals with residents or clients [19–22], and taking a multidisciplinary approach and providing ongoing training, team meetings and supervision to reinforce the approach on a daily basis [22]. One of the main challenges to susccessfully implementing programs has been compliance by staff [6].

The aims of this paper are to describe the development and components of reablement program for residential aged care – LifeFul; and conduct a feasibility study of the evaluation of LifeFul examining recruitment and retention; fidelity and adherence; acceptability, enablers and barriers; and suitability of outcome measures.

## Methods

### LifeFul intervention development

LifeFul was developed based on the MRC framework for the development of complex interventions [23]. The main steps were:

- Identification of the evidence base through
  (i) A literature review of reablement programs
    In summary, most randomized controlled trials demonstrated that reablement programs were successful in improving care recipient’s health [5, 7–9] reducing need for care or improving activities of daily living [11, 12], and were cost effective [24]. One trial was unsuccessful in reducing risk of death or permanent residential care [10]. Importantly, there have been few trials that have specifically targeted persons with dementia [6, 7]. In one study a reablement program improved overall function for cognitively intact residents but not for those who were cognitively impaired [6]. Reablement to date has focused primarily on physical and daily function but has not emphasized engagement with social and recreational activities to improve quality of life.
    There is a need for a reablement program focusing on all aspects of health and/or social care related quality of life and which specifically caters for residents with cognitive impairment and dementia.
  (ii) A systematic review of programs to change staff behavior in order to improve resident outcomes in residential aged care [25]. This review could not identify any intervention component, or combination of components targeting staff, which was more likely to result in improvement in outcomes in residents, it did however show that the few studies that used theory as part of program design was more likely to be successful in improving resident outcomes.
- Identification of relevant theory such as through a review of organization change literature such as fundamental principles in organizational change management in implementing effective changes [26, 27].
- Developing and describing the intervention.

A workshop was conducted with consumers, aged care providers, clinicians and academics to identify important elements and components of a sustainable reablement focused model of care. Initial meetings and ongoing discussions were undertaken with the leadership teams at participating facilities, senior executives and staff regarding program design and implementation.

#### Components of LifeFul

**Fig. 1:**
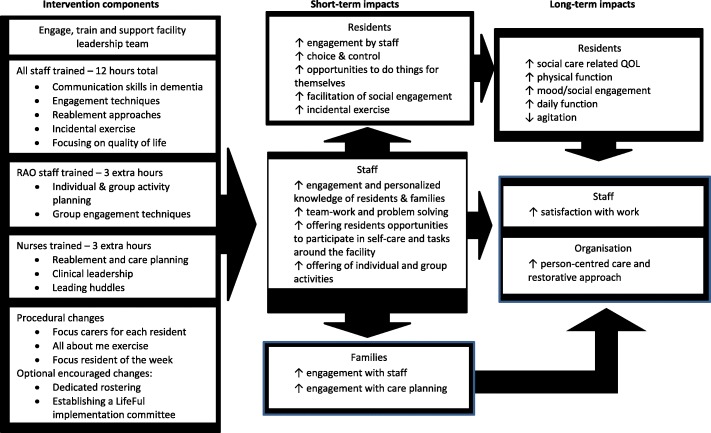
Lifeful program logic

The program logic for LifeFul was developed based on a) b) and c) above and is shown in Fig. 1.

The aims of the program are to improve social-care related quality of life and increase person-centred approaches to care by supporting staff to better engage with residents and be more individualized and enabling during social, physical, recreational and daily activities. By improving engagement of residents, we think that residents will experience better overall quality of life, as well as increased, physical and daily function, mood and wellbeing, and decreased agitation. In addition, staff will experience greater work satisfaction and improvement in delivering person-centred care. The philosophy underpinning the intended staff behaviour was person-centred care. Person-centred care in dementia care aims to maintain and nurture ‘personhood’ in dementia recognising the person’s identity, preferences and individual circumstances [28]. Person-centred care improves the quality of life of aged care residents [29]. Bandura’s social learning model and Kotter’s eight-step model for change were the key theories underpinning staff behaviour change [27, 30].

#### Engagement and supporting facility managers

The literature suggests that facility leadership has a key role in changing aged care workplace culture and in research projects [31]. Facility leaders (managers and deputy managers) were engaged and supported to facilitate organizational change.

Researchers met with facility managers and their leadership teams before LifeFul commenced at each site to discuss the program, and to understand needs and challenges at each site. The program was tailored to each site (e.g. one site had a psychosocial history sheet already; we adapted this rather than introducing a new form). Monthly tele-conferences with each site throughout the 12 months program helped monitor progress and barriers which were jointly addressed by the team. For instance, one site asked for an organization-endorsed list of activities around the home which residents were “allowed” to do, as there were misperceptions that policies existed that prevented residents from engaging should not be involved in housekeeping activities (e.g. setting tables). At another site staff struggled to execute the focus resident of the week (handover procedure). Based on feedback the handover form and procedure were changed substantively and reintroduced at the following training session.

#### Focus Carers and dedicated rostering

Each resident was allocated to a staff member (focus carer) who had a minimum of three shifts per week. The facility manager and leadership team aimed to match the focus carer with each resident based on the resident’s preferences, interests, gender, religion or cultural background and clinical care needs. The role of a focus carer was to specifically develop a good relationship with their focus resident, to get to know them and their social and activities needs. They were encouraged to get to know the resident’s family and friends, and work with them collaboratively to improve their resident’s quality of life; as well as advocate for the residents during handovers, case conferences, family meetings and care planning. This did not preclude all other staff from getting to know the resident.

There is some evidence that consistent assignment can improve staff-resident relationships and some clinical outcomes [32]. In two facilities, dedicated rostering was implemented. This involved rostering the same group of care staff consistently within the same geographical unit that consisted of between 12 and 20 residents in the facility. Consistent rostering allowed staff and residents to get to know each other better, and for staff to work as a team. In the third facility, one unit already had dedicated rostering, the remaining two units continued to rotate staff across units.

#### All about me

The purpose of the All About Me document was to facilitate staff getting to know their residents better. Focus carers were trained to obtain a brief psycho-social history of their resident and then collaborate with the residents to complete a visual representation of their resident’s social and activities needs and to set achievable goals (see Additional file 1: Appendix A for a fictitious example). These documents were placed within resident’s rooms (e.g. inside their wardrobe doors) and copies were easily accessible in staff rooms.

#### Handovers – Focus resident of the week

Focus residents of the week were introduced to improve team communication about residents’ psychosocial needs, focus on quality of life and clinical care, and to reinforce reablement and relationship-focused practices.. Facility managers selected a resident to focus on each week. Care teams set goals based on the resident’s goals relating to recreational, physical, and independence promoting activities so that residents had their ‘Best Week’ possible. Teams were to discuss the focus resident of the week briefly during every handover to ensure that every staff member got to know the resident better and contributed to implementing goals.

#### Staff training

All staff were provided with four, three-hour training sessions (12 h in total) over a period of 12 months, with sessions spaced 3 months apart (See Additional file 1: Appendix B). The training program was developed to be interactive, experiential and to support engagement with residents and introduce a reablement approach. As part of these 12 h, activity officers received discipline specific training on tailoring individual and group activities and lifestyle leadership, and nurses received discipline specific training on reablement and planning, clinical leadership and leading handovers.

Training sessions focused on the following:

- Session One - understanding resident’s psychosocial history and needs, goal setting, tailored approaches to care and activity engagement particularly for people with dementia, communication skills and completing the All About Me.
- Session Two - reablement approaches, creating opportunities to exercise choice and control, friendships and community in aged care, dementia and behaviour and focus resident of the week.
- Session Three - incidental exercise, reinforcement of skills from previous sessions, and staff self-care.
- Session Four - music, play and sustaining LifeFul.

To accommodate staff from various shifts attending the training program each session was run multiple times (Session 1 ran 9 times, Session 2 ran 11 times, Session 3 ran 9 times, and Session 4 ran 8 times).

### Feasibility study

This is the next phase of complex intervention development according to MRC guidelines.

#### Evaluation

The study has been approved by the University of Sydney’s Ethics Committee (2015/910) and is registered with the Australian New Zealand Clinical Trials Registry (ACTRN12616000070437).

#### Setting

The feasibility of LifeFul was evaluated within three aged care facilities in regional and metropolitan New South Wales, in two facilities we worked with the whole facility, in one facility we worked in three of six units. To be eligible facilities has to be government accredited, not enrolled in another intervention study and executive leadership and site leadership has to be willing to participate. The program was rolled out at unit level within facilities as it was not feasible to implement systemic practice change with some residents, so all residents were included in the intervention.

#### Study participants and recruitment processes

##### Staff recruitment

LifeFul training and practice change was mandatory for all care staff (i.e., care staff, registered and enrolled nurses (RNs and ENs), activity officers, allied health and managers) who worked on units in which LifeFul was implemented. Staff were reimbursed for attending training. However, participation in the evaluation was voluntary. Staff who joined the facility after baseline were invited to subsequent training and to participate in the evaluation at 12 months. Information about the study and consent forms were given to staff by the facility manager.

##### Resident recruitment

Residents and their families were informed about the program through posters, an information session and individual letters, and discussion at resident and family meetings. All permanent residents in participating facilities or units were invited to participate in the evaluation of the program (*N* = 146)*.* Information statements and consent forms were given to residents or posted to the person responsible by the facility manager. Written consent was sought from residents where possible. When the resident was not able to provide written consent due to cognitive or mental health related impairments, verbal consent was sought and written consent was obtained from their person responsible. We anticipated that 50% of 146 residents (i.e., *N* = 73) would consent to the evaluation of the program.

Residents who entered facilities and units after the commencement of the program were not recruited into the evaluation component.

#### Design

The evaluation used a within group pre-post intervention design. Mixed methods were used with quantitative and qualitative data collected. Evaluation occurred at 0 and 12 months for all sites.

#### Outcomes

##### Recruitment and retention

The number of staff and residents agreeing to participate in the study from those eligible within participating facilities were recorded. The number and reasons for dropouts from the study were also recorded.

##### Fidelity and adherence

The number of staff who attended each training (and those who did not) and training components delivered during training were recorded. An audit was conducted of whether each resident was allocated a Focus carer, completion rates of resident’s All About Me and resident of the week.

##### Acceptability, enablers and barriers

These were determined through evaluations completed by staff at the end of each training session, monthly tele-conferences with leadership teams, and focus groups and interviews at 12 months with staff and managers.

##### Suitability of quantitative measures

Outcome measures for residents and staff are listed in Table 1.

**Table 1:** Outcome Measures for Residents and Staff

Data	Instrument	Descriptor	Completed By			Time Point	
			Resident	Staff	Researcher	*T* = 0	*T* = 12
Resident’s Everyday Experiences							
Resident’s Autonomy, Control & Quality-of-Life	Adult Social Care Outcomes Toolkit (ASCOT): Care home interview schedule (CHINT-3); Care Home Resident Interview Schedule (CHResidentINT3); Care Home Observation Schedule (CHOBS3) [34]	Interview with resident and staff, and observation. ASCOT measures 8 domains: control of daily life; personal cleanliness and comfort; food and drink; personal safety; social participation and involvement; occupation; accommodation cleanliness and comfort; and dignity). Each domain is scored from 0 (high needs) to 3 (no needs) and is weighted to provide a final current QOL score. Inter-rater reliability: *r* = 0.618 (*p* < 0.001). Internal reliability Cronbach’s alpha = .71(Netten et al., 2010)	x	x	x	x	x
Resident’s Social Engagement	Social Identification and Satisfaction (SIS) [35]	By interview. SIS measures social identification and integrity and consists of 17 items organized into three subscales: social identification, satisfaction with lounge and satisfaction with life in the home. Each item is rated from 1 (completely disagree) to 7 (completely agree). Reliability Cronbach’s alpha = .70.	x			x	x
Physical Function	Short Physical Performance Battery (SPPB) (Guralnik et al., 1994) [36]	By interview. Battery consists of 3 tests: balance test, gait speed test and chair stand test. ICC = 0.82; reliability of gait speed test ICC = 0.79; reliability of chair stand test *r* = 0.80; reliability of tandem balance test is low (*r* = 0.22)(Puthoff, 2008)	x			x	x
Depression	Cornell Scale for Depression in Dementia (Alexopolous et al., 1988) [37]	19 items rated on severity based on interview with resident and staff. Each item is scores from 0 (absent) to 2 (severe) and a total score > 7 suggest high probability of clinical depression. It has internal consistency Cronbach’s alpha = 0.84; inter-rater reliability range: 0.67–0.74	x	x	x	x	x
Daily function	Disability Assessment for Dementia (DAD)(Gelinas et al., 1999) [38]	Informant-complete: 40 items which measures basic activities of daily living, instrumental activities of daily living and leisure activities. Each item is categorized into cognitive dimensions of initiation, planning and organisation and effective performance. Each item can be scored as yes (1), no (0), or not applicable. Test-retest reliability: .96; inter-rater reliability: .95 and internal consistency Cronbach’s alpha = .96.		x		x	x
Agitation	Cohen-Mansfield Agitation Inventory (CMAI) (Cohen-Mansfield, 1989) [39]	By interview: 29 items measuring agitated behaviours in elderly person. Each item is rated on frequency from 1 (never) to 7 (several times an hour). Cronbach’s alpha 0.75–0.91(in different studies); test-retest r: 0.79–0.9; inter-rater correlations: 0.76–0.96		x		x	x
Satisfaction with Work	Nursing Home Nurse Aide Job Satisfaction Questionnaire (NHNA-JSQ) (Castle et al., 2007) [40].	NHNA-JSQ is a 21 item measure (each item rated from 1- *very poor* to 10 - *excellent*) and has seven subscales – (1) Coworkers (the relation that the person has with other workers in the facility), (2) Workplace Support (resources and demands of the job), (3) Work Content (the complexity and challenge of the work), (4) Work Schedule (time pressures), (5) Training (preparation required for position, (6) Rewards (benefits of the job) and (7) Quality of Care (how well nurse aides perceive residents are being cared for). The NHNA-JSQ has good internal consistency (Cronbach’s alpha > .74).		x		x	x
Person-Centred Care Approach	Person-Centered Care Assessment Tool (P-CAT) (Edvardsson et al., 2010) [41]	P-CAT is a13 item measure (1 = *disagree completely* to 5 = *agree completely)*. The P-CAT measures the degree to which staff engage in person-centred care and has three subscales – personalising care (the degree to which staff and the organisation adhere to person-centred care), organisational support (the degree to which the organisation supports staff to engage in person-centred care) and environmental accessibility (the degree to which residents can access their immediate environment). The P-CAT has good internal consistency (Cronbach’s alpha = 0.84) and high retest reliability (*r* = .7–.9).		x		x	x

##### Data analyses

SPSS software was used for analyses. Descriptive statistics were produced for resident and staff demographics, recruitment and retention, fidelity and adherence and quantitative measures. Quantitative measures were examined for ceiling and floor effects, and completion rates. Multilevel linear models were used to examine the change in outcomes between baseline and 12 months. These models take into account correlations between repeated measures. These analyses took an intention-to-treat approach, as multilevel linear models can handle missing data at different time points.

In order to examine acceptability, enablers and barriers we utilized qualitative content analysis [33] to analyze exit interviews, focus groups and meeting minutes. All these data were transcribed. Based on the transcriptions, one author (SV) systematically coded recurrent themes, these were checked by a second author (LFL) and discrepancies resolved through discussion.

## Results

### Recruitment and retention of residents and staff

All 146 residents living at baseline in the selected units were approached to be part of the evaluation of the program and 80 residents (54.8%) consented to participate. Resident demographic information is presented in Table 2. At 12 months, 11 (13.8%) residents were deceased, we were collect dataed from informants on the remaining 69 (86.3%) residents however were only able to interview 67 (83.8%) residents due to increased cognitive impairment.

**Table 2:** Resident Demographics at baseline (*n* = 80)

Variable	Mean (SD, range) or number (%)
Age	87.6 (7.5, 63.6–98.8)
Female	64 (80.0%)
Born Overseas	9 (11.3%)
Marital Status	
Single	9 (11.3%)
Widowed	50 (62.5%)
Divorced	5(6.3%)
Married/Partnered	16 (20%)
Days lived in facility	1042.8 (77; 5–3804)
No of Medical & Psychological Diagnoses	8.1 (3.2, 1–15)

A total of 157 staff were approached to be part of the evaluation at baseline and 104 staff (66.2%) participated at baseline. At 12 months, 123 of these staff were still working at these facilities and 85 (81.7% of baseline participants) participated in the evaluation. We did not manage to collect data on how many new staff joined the program through the year, however 36 additional staff participated at 12 months. A total of 140 staff participated in the evaluation over the year. Staff demographics are presented in Table 3.

**Table 3:** Staff Demographics (*n* = 140)

Variable	Mean (SD, range) or number (%)	*N* Missing
Age	42.8 (12.5, 18–67)	13
Female	124 (89%)	1
Born in Australia	115 (82%)	13
Years of Education	12.9 (2.8, 8–21)	73
Highest Education		59
School Certificate	22 (15.7%)	
Trade Certificate	30 (21.4%)	
Undergraduate Degree	28 (20.0)	
Post-Graduate Qualification	1 (0.7%)	
Position at Organisation		1
Registered Nurse	18 (12.9%)	
Activity Officer	11 (7.9%)	
Care Staff	97 (69.3%)	
Pastoral Care	1 (0.7)	
Care Manager	4 (2.9%)	
Kitchen Hand	5 (2.6%)	
Physiotherapist Assistant	2 (1.4%)	
Administrative Staff	1 (0.7%)	
Hours Worked (per week)	28.2 (8.0, 10–40)	17
Years working in aged care facilities	5.5 (5.7, 0.1–29)	16

### Fidelity and adherence

All residents who consented to participate in the evaluation process of the study were allocated a focus carer. Sixty-nine residents (86.3%) had an All About Me completed. All About Me’s were not completed because some residents did not want them (2.5%) and some staff members had not completed them for their allocated resident (3.75%).

Thirty-six residents (45%) had been focus resident of the week. Reasons that resident’s had not been focus residents of the week were that some staff did not understand how to complete the procedure, and the handovers were not scheduled and implemented by facilities’ leadership teams.

The attendance of eligible staff at each session was: session 1–88% (110 of 125), session 2–61.79% (76 of 123), session 3–87.80% (108 of 123) and session 4–76.07% (89 of 117). Staff missed training due to illness, annual leave, forgetting, not knowing about training and having to cover for direct care staff.

### Acceptability, barriers and enablers

#### Acceptability – Post training session evaluations

The majority of staff described the training material as easy to understand (99.28%), relevant to their workplace (99.28%) and the training helped with understanding (97.12%). Written comments suggested that staff enjoyed the activities and most of the content, however found a few concepts difficult to understand (e.g. Maslow’s hierarchy of needs, basic task analysis, stages of dementia).

#### Acceptability – Exit interviews and focus groups

Interviews and focus groups suggested that staff found the program acceptable, many staff describing benefits of the program. Staff said that LifeFul *helped them to build better relationships with residents* by taking time to get to know them, listening to them, and relating to them on a personal level. This led to increased understand the residents’ behaviors and needs.

*“Being able to have closer insight into the client and better understanding of why people do the things they do and why, they react differently with different staff”*

*“This has given me more understanding about residents on a more personal level – more knowledge how to relate to residents”*

Staff also described how LifeFul *helped them develop their skills* by giving them specific skills in improving their communication and presenting activities.

***“*** *Learning new methods to make residents lives happier/better. Fresh ideas.”*

Staff also reported that LifeFul encouraged them to *be more creative* and think laterally when solving problems at work.

***“*** *Being creative also helps with problem solving which is really important in a dementia unit.”*

Staff members noticed *positive changes in the units* they worked in. Residents were more settled as a consequence of implementing some of the strategies from LifeFul, for instance by increasing family involvement and accepting family as part of the community rather than perceiving them as visitors. Staff began involving residents in the daily tasks of the unit (e.g., cleaning tables, folding clothing protectors, helping to push trolleys).

*“The ‘All About Me’ sheets gave us a starting point. An insight into the resident’s personality. What staff initially saw as uncooperative they now saw as proud and independent. Physical outbursts are no longer viewed as unpredictable or malicious. Incidents of hitting rarely happen now, and if they do, we understand why they happened and what our response should be. Staff are building up a level of trust with her and seeing her for who she is, and not just as unmanageable negative behaviors.”*

### Barriers

*Having limited time at work, or a long list of work tasks* meant that staff found it difficult to spend time getting to know residents and complete new program procedures.

*“Found it stressful, I had two focus residents plus I had to do that and make sure I talked to the families, residents and make sure that I do my work.”*

When there was a *small number of staff on each unit* (sometimes only 1) the lack of time seemed to be exacerbated, as staff didn’t have team-mates to help solve clinical problems, discuss daily stressors, for motivation and to change the unit atmosphere. This was an issue in low care units.

*“If there were more staff, they could be more activities, more social interaction, getting to know the residents. It would also give someone for the care staff to bounce ideas off”*

*Negative or ambivalent attitudes of staff* towards the program were also described as a barrier. Some staff did not see program implementation as their responsibility, did not prioritize or see value in implementing the program, or did not want to change their usual care practices.

*“No matter where you go you will have a small amount of people if something new is going to be implemented it’s always going to be negative.”*

Some staff also *did not understand the new procedures* (All About Me, Best Week Handover), even though they had received training. They were unsure about whose responsibility it was to complete the procedures, as well as the correct way of filling out the documents.

*“Some staff suggested more clarity around forms; specifically the handovers as they were interpreted differently to what was initially intended.”*

Staff found it more difficult implementing the program with residents with *later stage dementia*. They described it being more difficult to communicate, obtain information, set goals for, and motivate these residents.

In two units where staff were rotated in and out of those areas every 3 months (i.e. where dedicated rostering was not implemented), the program appeared to have the least impact based on staff feedback, even though All About Me sheets and focus resident of the week were completed. Staff found it difficult to spend time with their focus resident when working in a different area, and were not motivated to work on long-term goals for residents as they could be rotated out of that area before being able to meet those goals.

Staff, facility managers and executive managers all commented on the importance of the *facility leadership team* (manager and unit or deputy managers) in implementing the program. Some staff perceived that the program was not supported sufficiently by their manager. We also observed that the program stalled if facility leadership did not continue to motivate staff and complete administrative and logistical aspects of the program (e.g. assigning focus carers, scheduling timetable of focus residents of the week), as well as role modelling behavior (e.g. attending Best Week handovers).

*“Management need to support staff at different stages of building that relationship with carers, such as, starting to do the All About Me sheets, or having a focus resident at handover.”*

### Enablers

Specific staff members were described as acting as informal or formal *program LifeFul champions.* On some units, a key person or persons took their own initiative, in one facility staff were selected by facility managers and asked to support their colleagues. These champions facilitated program implementation through organization, education, motivation, resource development (e.g. obtaining materials for activities), and role modelling.

*“Thank God for X. She helped me and all of us. If it wasn’t for her I wouldn’t have finished”*

Units where staff were already *working well as a team*, or where team work and morale improved through LifeFul, reported better success in implementing practices changes beyond the procedural aspects of the program.

### Suitability of quantitative measures for residents and staff

Residents’ scores on the outcomes measures at baseline and 12 months, and the results from multilevel linear models are reported in Table 4. On the Adult Social Care Outcome Toolkits (ASCOT) self-complete component at baseline and 12 months, 66 (82.20%) and 52 (76.47%) residents completed them respectively. Some residents did not complete the ASCOT self-complete component because of difficulties with communication and/or comprehension. On the informant component of the ASCOT, we obtained data for 76(95%) residents at baseline and all 69 (100%) residents at 12 months. Some staff informants were unable to score domain 1 (control) of the ASCOT at baseline as these residents were non-communicative. The majority of scores on ASCOT domains fell into the *No Needs* category (80.30–98.50%), suggesting the possibility of a ceiling effect on this tool.

**Table 4:** Resident outcomes at baseline and 12 months

Outcome Measure	Baseline M (SD, *N*)	12 Months M (SD, *N*)	Difference between baseline and 12 months (95% confidence interval)
Adult Social Care Outcome Toolkit ASCOT			
Domain 1 Control	0.79 (0.23, *N* = 76)	0.85 (0.21, *N* = 67)	b = − 0.05 (− 0.12 to 0.01)
Domain 2 Personal Hygiene	0.86 (0.14, *N* = 76)	0.88 (0.07, *N* = 67)	b = − 0.02 (− 0.04 to 0.01)
Domain 3 Food	0.79 (0.19, *N* = 76)	0.80 (0.18, *N* = 67)	b = − 0.02 (− 0.08 to 0.04)
Domain 4 Safety	0.69 (0.10, *N* = 76)	0.72 (0.00, *N* = 67)	b = − 0.03 (− 0.05 to − 0.01)
Domain 5 Social Participation	0.72 (0.17, *N* = 76)	0.76 (0.15, *N* = 67)	b = − 0.04 (− 0.07 to 0.00)
Domain 6 Occupational Engagement	0.79 (0.20, *N* = 76)	0.85 (0.18, *N* = 67)	b = − 0.05, (− 0.11 to 0.00)
Domain 7 Accommodation	0.85 (0.05, *N* = 76)	0.86 (0.00, *N* = 67)	b = − 0.01 (− 0.17 to 0.06)
Domain 8 Dignity	0.75 (0.12, *N* = 76)	0.78 (0.00, *N* = 67)	b = − 0.03 (− 0.54 to − 0.01)
ASCOT Total (SCRQoL)	0.84 (0.17, *N* = 76)	0.89 (0.12, *N* = 67)	b = − 0.05, (− 0.08 to − 0.02)
Short Physical Performance Battery (SPPB)			
Balance Test Total Score	1.63 (1.05, *N* = 52)	1.81 (1.09, *N* = 36)	b = 0.07 (− 0.20 to 0.34)
Gait Test Total Score	2.40 (0.95, *N* = 52)	2.64 (0.90, *N* = 36)	b = 0.06 (− 0.24 to 0.35)
Repeated Chair Stand Score	0.67 (1.06, *N* = 52)	0.81 (1.14, *N* = 36)	b = 0.12 (− 0.17 to 0.42)
Social Identification and Satisfaction Subscale (SIS)			
Social Identification	4.36 (0.96, *N* = 57)	4.30 (0.86, *N* = 50)	b = 0.04 (− 0.27 to 0.35)
Satisfaction with Lounge	4.79 (1.52, *N* = 57)	4.54 (1.34, *N* = 50)	b = 0.26 (− 0.18 to 0.70)
Satisfaction with Life in the Home	3.67 (0.72, *N* = 57)	3.66 (0.75, *N* = 50)	b = 0.11 (− 0.20 to 0.42)
Cornell Depression Total	7.12 (7.91, *N* = 80)	4.49 (4.30, *N* = 67)	b = 2.28 (0.77 to 3.79)
Cohen Mansfield Agitation Inventory (CMAI) Total	40.94 (14.31, *N* = 80)	40.76 (16.32, *N* = 67)	b = − 0.32 (− 3.74 to 3.10)
Disability Assessment for Dementia (DAD) Total	41.46 (27.45, *N* = 80)	50.83 (32.96, *N* = 67)	b = − 2.15 (− 10.61 to − 0.38)

On the Short Physical Performance Battery (SPPB), 52 (70.00%) and 36 (53.73%) participants were able to complete all three subscales at baseline and 12 months, respectively.

Some residents found it difficult to complete the seven-point Likert scale for the Social Identification and Satisfaction Subscale (SIS), we requested those residents respond *Yes* or *No* instead. At baseline and 12 months, 57 (71.25%) and 50 (74.63%) participants were able to complete all three subscales on the measure.

We obtained complete data on the three measures that were completed by staff on residents’ mood, behavior and daily functioning (i.e., Cornell Depression Scale, Cohen Mansfield Agitation Inventory – CMAI and Disability Assessment for Dementia – DAD) at baseline and 12 months.

Staff scores on the outcomes measures at baseline and 12 months, and the results from multilevel linear models are reported in Table 5. On the Nursing Home Nurse Aide Job Satisfaction Questionnaire (NHNA-JSQ) 104 (66.24%) and 95(77.24%) staff completed the outcome measure at baseline and 12 months, respectively. On the Person-Centered Care Assessment Tool (P-CAT), 98 (62.42%) and 95 (77.24%) staff completed the outcome measure at baseline and 12 months, respectively. Fewer staff completed the P-CAT than the NHNA-JSQ at baseline due to a procedural error in filling out the evaluation form.

**Table 5:** Staff Outcomes at baseline and 12 months

Outcome Measure	Baseline M (SD, *N*)	12 Months M (SD, *N*)	Difference between baseline and 12 months (95% confidence interval)
Nursing Home Nurse Aide Job Satisfaction Questionnaire (NHNA-JSQ)			
Co-Workers	7.64 (1.76, 104)	7.73 (1.57, 95)	b = − 0.22 (− 0.56 to 0.12)
Workplace Support	7.36 (1.44, 104)	7.62 (1.56, 95)	b = − 0.27 (− 0.61 to 0.06)
Work Content	8.76 (1.05, 104)	8.76 (1.05, 95)	b = − 0.01 (− 0.25 to 0.26)
Work Schedule	7.29 (1.61, 104)	7.38 (1.66, 95)	b = − 0.14, (− 0.56 to 0.28)
Training	8.17 (1.31, 104)	7.98 (1.60, 95)	b = 0.14 (− 0.18 to 0.49)
Rewards	6.19 (2.25, 104)	6.10 (2.28, 95)	b = 0.08 (− 0.35 to 0.50)
Quality of Care	8.58 (1.13, 104)	8.64 (1.19, 95)	b = − 0.13 (− 0.38 to 0.13)
Global Ratings	8.24 (1.53, 104)	7.96 (1.84, 95)	b = 0.19 (− 0.18 to 0.56)
Person-Centered Care Assessment Tool (P- CAT)			
Personalising Care	3.69 (0.75, 98)	3.79 (0.80, 95)	b = − 0.13 (− 0.30 to 0.05)
Organisational Support	2.76 (0.86, 98)	2. 69 (0.83, 95)	b = 0.06 (− 0.18 to 0.28)
Environmental Accessibility	3.18 (0.92, 98)	3.17 (0.87, 95)	b = − 0.01 (− 0.22 to 0.20)

### Changes over time

There was a reduction in residents’ depression symptoms (Cornell Depression), improved functioning (Disability Assessment for Dementia Scale) and improvement in the ASCOT domains of safety, occupational engagement, dignity and overall quality of life (ASCOT SCRQoL). There were no differences between baseline and 12 months on the SPPB Subscales, SIS subscales or the CMAI. See Table 4.

There were no changes over time on any of the staff outcome measures. See Table 5.

## Discussion

This study demonstrated that it is feasible to implement and evaluate LifeFul, a reablement and relationship-focused program to change staff care practices in residential aged care facilities. LifeFul changed some staff care practices and produced promising improvements in resident outcomes. However, there were challenges in implementation. Less than 80% of staff attended training. In the future we would work more closely with facility managers to ensure that mandatory training attendance was policed, that staff were given sufficient notice about training dates, and that staff were not pulled out of training to back fill for staff on leave. The original format for focus resident of the week handovers had low acceptability. We changed these so that care teams spent 1 week gathering information about residents and setting goals, and then wrote a psychosocial care plan the following week. LifeFul required ongoing motivation of staff, this might be achieved by better preparing and supporting the facility leadership team to lead the change required in the program, as well as appointing staff champions to assist with administration, execution and motivation.

There may be a ceiling effect on the proposed primary outcome measure of social care related quality of life (the ASCOT) which makes it less sensitive to changes as a consequence of the program. We identified no other suitable measure of social-care related quality of life in residential care. Increasing the scale of ASCOT items may minimize this ceiling effect, this is currently being tested by the research team that developed the scale. The ceiling effect may reflect the high quality of care provided in participating facilities, and may not be representative of Australian residential care facilities.

Strengths of this study are that we worked with organizations which supported the procedural changes required in LifeFul, the research team worked closely with facility management to monitor implementation and clarified, adapted and supported as needed. The training component was commended by staff as being interactive and engaging.

This feasibility study was not designed to produce generalizable results. Our small sample came from a non-representative group of three volunteer residential aged care facilities, all in regional areas with low proportions of residents from culturally and linguistically diverse backgrounds, and relatively low staff turnover (16.20% across two sites from the same organization). The pre-post uncontrolled design means that we cannot be certain that our intervention had a causal impact on observed resident outcomes. We also did not correct for multiple comparisons, or control for age, gender or other characteristics.

## Conclusions

In conclusion, these results suggest that it is feasible to deliver and evaluate LifeFul. A fully-powered controlled trial, including an economic analysis, is required to see if the program can improve resident outcomes.

## Additional file

Additional file 1: Appendix A: Fictitious sample of All About Me sheet and Appendix B: Description of LifeFul training program. (DOCX 603 kb)

## Abbreviations

ASCOT: Adult social care outcome toolkits
CDS: Cornell depression scale
CMAI: Cohen mansfield agitation inventory
DAD: Disability assessment for dementia
EN: Enrolled nurses
NHNA-JSQ: Nursing home nurse aide-job satisfaction questionnaire
P-CAT: Person-centered care assessment tool
RN: Registered nurses
SIS: Social identification and satisfaction subscale
SPPB: Short physical performance battery
