# Supplementary material for: Feasibility of LifeFul, a relationship and reablement-focused culture change program in residential aged care
Source: BMC Geriatr. 2018 May 31;18:129. doi: 10.1186/s12877-018-0822-3 (PMC5984457; doi:10.1186/s12877-018-0822-3)
Supplement: Supplementary file 1 — Appendix A: Fictitious sample of All About Me sheet and Appendix B: Description of LifeFul training program. (DOCX 603 kb) [file 12877_2018_822_MOESM1_ESM.docx]

**Appendix A LifeFul Training Program, Learning Objectives and Training Activities**

| **Session number and**  **description** | **Learning Objectives** | **Main Training Activities** |
| --- | --- | --- |
| Session 1 - Introduction to LifeFul, being a focus carer, stages of dementia and All About Me and goal setting | - Understand how relationship based reablement (and being a focus carer): (i) improves a resident’s physical, social and psychological quality of life (QOL)and (ii) enhances staff’s workplace satisfaction and team cohesion - Construct an individualised “All About Me” document including life history and goals set by residents and families - Use various models (e.g., Maslow’s Hierarchy of Needs) and frameworks ( e.g., stages of dementia and the associated symptoms) to understand a resident to provide them with individualised and personalised care - Apply effective communication strategies to empower residents and staff and to improve social connectedness | 1. Discussion on the positive and negatives aspects of their roles and which of these LifeFul will improve 2. Demonstrating how skills learnt from session are used to complete resident’s All About Me document 3. Discussion on models pertinent to understanding a resident’s needs including, Maslow’s Hierarchy of Needs, stages of dementia and applying this information to personalise care for their focus resident 4. Generating an individualized activity ( group and/or individual) for the resident taking into account a person’s cognition 5. Practicing effective listening and communication skills (good and bad listening, using effective listening techniques, interviewing resident’s on their life history) 6. Discussion on how to involve families in care |
| Session 2 – teamwork,  reablement, introducing ‘resident of the week’ and huddles | - Understand the impact of deconditioning on a resident’s physical, social and psychological wellbeing; and use strategies such as promoting autonomy, choice and control, task analysis, and environmental modifications to promote reablement - Recognize how ostracism can contribute to decreased social connectedness and implement strategies to promote social participation, interactions and improve QOL for residents - Understand how stages of dementia impact on resident’s support needs for independence, friendship and autonomy - Implement effective strategies to improve team cohesion, communication and team satisfaction, in particular, team huddles (handovers) | 1. Exploring effects of living in a nursing home, particularly deconditioning and how it is maintained 2. Roleplay activity and discussion of providing care that creates dependence vs care that creates interdependence (including discussion of environmental strategies, task analysis of an ADL, setting a reablement target, setting up unstructured activities) 3. One participant in a group is excluded temporarily from a ball game to illustrate psychological effects of ostracism and how this contributes to deconditioning. 4. Creating friendship diagrams for residents within units and how to encourage friendships between like- minded residents 5. Introducing supplement to handovers that monitors focus resident’s participation in individual and group activities. |
| Session 3 – incidental  exercise, behavior analysis, revisiting goal setting | - Recognize the benefits of physical activity on physical, social and psychological QOL and implement strategies (i.e.,standardised instructions to encourage independent mobilization; balance and strength exercises) to promote physical movement in resident’s daily lives - Understand how behaviour change in dementia is affected by situations, emotions and belief and use a variety of strategies to manage behaviours - Improve goal setting (short term & long term) with residents by utilizing a variety of sources including: care plans, All About Me, huddles, feedback from staff and families - Recognize the symptoms of burn out and grief in staff, and be aware of resources and strategies to manage these (i.e., self-care, mindfulness). | 1. Activity that requires staff to estimate how many steps older adults take to recognise physical activity needs. 2. Demonstrate standardized instructions to improve confidence in mobilisation. Ask participants to practice it in pairs 3. Discussion of incidental exercise and how to encourage residents’ involvement 4. Demonstration and practice of how to safely practice balance and strength exercises with residents 5. Analyzing a behaviour to improve management and its consequences (ABCDE mnemonic) 6. Generating short term and long term goals for residents using Maslow’s Hierarchy of needs 7. Discussion on what is grief and loss, how to cope with it and seeking help if needed 8. Understanding triggers of stress at home and at work and implementing a self-care plan, including the practice of mindfulness |
| Session 4 | - Recognize how music can affect mood and elicit memories; and use strategies including play lists, moving along with music and incorporating music into resident’s daily life to better manage resident’s mood and behaviours - Understand the approach of a playful state of mind, its benefits on physical, social and psychological well-being including in the workplace - Understand how personal and organisational values can influence decision making, interpersonal interactions, including the status one gives to a person and apply strategies to decrease potential power differentials and empower individuals within relationships - Evaluate the program by identifying the successes and challenges, and identify any necessary modifications or strategies to maintain its success | 1. Discussion on how music assists to reminisce and elicits emotions 2. Generating strategies to create a personalised play list for resident 3. Discussion of how to use music to manage mood, how to engage residents in music with varying cognitive abilities with the use of movement 4. Group activity, charades, to demonstrate importance of play 5. Discussion on which activities constitutes play, the approach of play and its benefits 6. Discussion about society’s values (using the Lifeboat ethical dilemma) 7. Group exercise in which members of a group take on a low or high status and must come to a decision about a holiday 8. Discussion on status of residents and how to promote their status 9. Reflecting on the successes and challenges, how to problem solve these. 10. Discussion on how to improve the program and maintain the momentum within their facility |
| Alternative Session 2 –  Nurse training (registered and enrolled nurses) – being clinical leaders as part of LifeFul | - Understand the pivotal role of a good leader, how it differs from management and the qualities and skills required to drive change within the organisation - Understand the impact of deconditioning on a resident’s physical, social and psychological wellbeing; and use strategies such as promoting autonomy, choice and control, task analysis, and environmental modifications to promote reablement - Recognize how ostracism can contribute to decreased social connectedness and implement strategies to promote social participation, interactions and improve QOL for residents - Implement effective strategies to improve team cohesion, communication and team satisfaction, in particular, team huddles (handovers) | 1. Drawing the facility’s organizational chart and examining how they see themselves as leaders, what do they value as a clinical leader and how can they overcome challenges they face as clinical leaders 2. Exploring effects of living in a nursing home, particularly deconditioning and how it is maintained 3. Roleplay activity and discussion of providing care that creates dependence vs care that creates interdependence (including discussion of environmental strategies, task analysis of an ADL, setting a reablement target, setting up unstructured activities) 4. Creating friendship diagrams for residents within units and how to encourage friendships 5. Introducing supplement to handovers that monitors focus resident’s participation in individual and group activities. |
| Alternative Session 2 –  Recreational Activity Officer – being Lifestyle leaders as part of LifeFul | - Understand the pivotal role of a good leader, how it differs from management and the qualities and skills required to drive change within the organisation - Recognize that activities can have therapeutic benefits (including managing difficult behaviour) and implement strategies such as an individualised activity calendar to improve the physical, social and psychological QOL of residents, particularly those with dementia - Understand the impact of deconditioning on a resident’s physical, social and psychological wellbeing; and use strategies such as promoting autonomy, choice and control, task analysis, and environmental modifications to promote reablement - Recognize how ostracism can contribute to decreased social connectedness and implement strategies to promote social participation, interactions and improve QOL for residents - Implement effective strategies to improve team cohesion, communication and team satisfaction, in particular, team huddles (handovers) | - Drawing the facility’s organizational chart and examining how they see themselves as leaders, what do they value as a lifestyle leaders and how can they overcome challenges they face as lifestyle leaders - Exploring effects of living in a nursing home, particularly deconditioning and how it is maintained - Roleplay activity and discussion of providing care that creates dependence vs care that creates interdependence (including discussion of environmental strategies, task analysis of an ADL, setting a reablement target, setting up unstructured activities) - Discussion of how activities can be therapeutic and can lead to health benefits for the resident - Incorporating the resident’s preferences of activities in the group activity calendar - Modifying groups and roles within groups to cater for a variety of cognitive levels (including those with dementia) - Generating strategies to increase socialization between residents, particularly at similar stage of dementia - Creating friendship diagrams for residents within units and how to encourage friendships - Introducing supplement to handovers that monitors focus resident’s engagement in individual and group activities. |

**Appendix B** **Example All About Me document**
